# Association between low carbohydrate diet (LCD) and sleep quality by mediating role of inflammatory factors in women with overweight and obesity: A cross‐sectional study

**DOI:** 10.1002/fsn3.2584

**Published:** 2021-09-28

**Authors:** Atefeh Tavakoli, Atieh Mirzababaei, Khadijeh Mirzaei

**Affiliations:** ^1^ Department of Community Nutrition School of Nutritional Sciences and Dietetics Tehran University of Medical Sciences (TUMS) Tehran Iran

**Keywords:** inflammatory factors, low carbohydrate diet, obesity, overweight, sleep quality

## Abstract

Poor sleep quality can lead to increased obesity. Low carbohydrate diet (LCD) is considered as an approach for sleep quality and obesity improvement. The aim of this investigation is to evaluate the relationship between LCD and sleep quality with the mediatory effect of inflammatory markers including transforming growth factor‐β (TGF‐β), high‐sensitivity C‐reactive protein (hs‐CRP), total antioxidant capacity: In our cross‐sectional study, 304 obese women aged from 19 to 50 years were enrolled. Body mass index (BMI) in these women ranged from 25.2 to 48.3 kg/m^2^. LCD score was assessed by a 147‐ item semi‐quantitative food frequency questionnaire (FFQ). The Pittsburgh Sleep Quality Index (PSQI) was used to assess the sleep quality. FFQ and PSQI questionnaires are completed simultaneously by the participants. Biochemical indicators (inflammatory markers) were measured and anthropometric components were evaluated. The relationship between sleep quality and LCD with quantitative variables was assessed by independent sample *t*‐test and with qualitative variables by chi‐square test. Binary logistic regression was used to estimate confounding variables including age, job, stress, weight to investigate the relationship between LCD and sleep. Following of LCD had a significant negative relationship with PSQI score. It can be said that with increasing LCD adherence, the possibility of poor sleep quality decrease (OR = 0.43, 95% CI = 0.19–0.94,*p* = .03). It was also showed, hs‐CRP (OR = 0.61, 95% CI = 0.3–1.21, *p* = .16) and TAC (OR = 0.6, 95% CI = 0.25–1.4, *p* = 0.24), eliminated the significance of the association and it is possible that they play a mediating role in this relationship Following the LCD can have a positive effect on improving PSQI scores by reduction in inflammatory markers levels.

## Graphical abstract

Poor sleep quality can lead to increased obesity. Low carbohydrate diet (LCD) is considered as an approach for sleep quality and obesity improvement. The purpose of this study is to examine the association between adherence of LCD and sleep quality by the mediatory effect of inflammatory markers including high‐sensitivity C‐reactive protein (hs‐CRP), transforming growth factor‐β (TGF‐β), total antioxidant capacity (TAC) and interleukin‐1β (IL‐1β) in women with overweight and obesity.

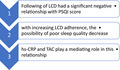

## 1 INTRODUCTION

Obesity as a serious public health concern has risen around the world during the last decades. Excess energy intake, which is usually shown as a positive energy balance that leads to an increase in body fat, can be considered as a nutritional factor for weight gain(Apolzan et al., 2014). Obesity is associated with chronic diseases incidence including cardiovascular disease, diabetes, cancers such as breast and endometrial cancer in women (Hedley et al., 2004; Hu, 2003).

Unhealthy dietary patterns and poor sleep quality can increase the obesity trend (Hu, 2003). According to the results of several studies, there is a relationship between sleep duration and the incidence of obesity (Patel, 2009; Patel & Hu, 2008). Lack of adequate sleep or short sleep duration affect energy intake and elevate it, notably from snacks (Grandner et al., 2010). There are many approaches to define sleep quality, such as polysomnography and actigraphy; according to them, sleep quality is characterized by sleep‐onset latency (SOL) and sleep efficiency (SE). Long SOL (generally>20–30 min depending on age) and low SE (generally <85%) typically characterize poor sleep. Sleep quality, also, may be defined by the amount of rapid eye movement (REM) and slow wave sleep (SWS), which the person gets during sleep. SWS has a stronger function in creating deep sleep (Dijk, 2009; Golem et al., 2014). Studies have shown that SWS and REM as two stages of sleep are inversely related to the consumption of fats and carbohydrates (Shechter et al., 2012). Increased carbohydrate intake can affect REM sleep and SWS primarily. Phillips et al. examined the association of low‐carbohydrate/high‐fat (LC/HF) and high‐carbohydrate/low‐fat (HC/LF) diets with sleep quality. HC/LF diet reduced SWS compared to a LC/HF diet. REM increased with both dietary interventions but showed a greater increase after consuming a HC/LF diet. ( Phillips et al., 1975). Yajima et al. (2014) have shown that eating a HC meal increased carbohydrate oxidation, and SWS was markedly reduced as a consequence of HC meal. The researchers acknowledged that effects of a HC meal on sleep quality are associated with decreased melatonin synthesis. Reducing carbohydrate intake, especially before bedtime, leads to decreased secretion of inflammatory markers, which in turn resulted to increased expression of clock‐genes and improved sleep regulation (Milagres et al., 2014; Porter & Horne, 1981; Tanaka et al., 2013). Weight loss can decrease the amount of adipose tissue in healthy obese person who do not have any cachectic diseases such as cancer and do not live in catabolic conditions due to disease or surgery and may be considered as an approach of inflammatory marker level such as interleukin‐6 (IL‐6) an hs‐CRP reduction (Sharman & Volek, 2004). A study reported that adiponectin, which is known as an anti‐inflammatory agent, was negatively correlated with the glycemic load and carbohydrate content of the diet and (Yannakoulia et al., 2003). Recent investigations have found that tumor necrosis factor (TNF) and IL‐1 were associated with sleep deprivation (Motivala & Irwin, 2007). Decreased leptin and increased ghrelin levels are related to short sleep duration, and obesity as a chronic inflammation may be associated with insufficient sleep (Spiegel et al., 2004; Taheri et al., 2004).

Weight loss by dietary restriction may improve sleep quality by reducing sleep fragmentation and alleviating sleep‐disordered breathing. On the other hand, good sleep quality and longer sleep duration may promote weight loss by reducing appetite (Kalam et al., 2021) problems such as poor sleep quality, which are more common in women than in men; women are also more likely than men to have diet‐related problems such as cravings for certain foods that can lead to obesity in women (Maddahi et al., 2020) Women are one of the most sensitive groups in society, especially in developing societies, and the incidence of inflammatory conditions such as viral and respiratory infections is higher in women than in men (Tavakoli et al., 2021), so we chose this group as the target group.

As far as we know the association of LCD adherence and sleep quality due to inflammatory factors has not been studied in any investigation, so we aimed to evaluate the relationship between LCD adherence and sleep quality that mediated by four inflammatory factors including total antioxidant capacity (TAC), hs‐CRP, IL‐1β and TGF‐β in obese and overweight women. Our hypothesis in this study is that following a low‐carbohydrate diet may be associated with poor sleep quality due to increased inflammatory markers in women.

## 2 METHOD

### 2.1 Study population

In this cross‐sectional study, 304 overweight and obese 19‐ to 50‐year‐old women were participated. Sample size was calculated based on the following formula: *n* = [(*Z* 1‐α + *Z* 1‐β) × √1‐ *r*2]/*r*) 2 + 2), where α = 0.05, β = 0.95, *r* = 0.25. All participants were randomly selected from those who referred to health centers in Tehran. Inclusion criteria indicated as: aged 19 to 50 years and being overweight or obese (BMI ≥25 kg/m^2^); exclusion criteria indicated as: pregnant or lactation or being at menopausal period, chronic diseases such as diabetes mellitus, kidney and liver diseases, cardiovascular diseases such as hypertension, addiction to drugs and alcohol, regular use of medications except contraceptives, body weight changes in the last year so that the weight changes were more than 5%, weight loss surgery, and weight loss diets. All women signed a consent form, and our study was confirmed by the local Ethics Committee of Tehran University of Medical Sciences (IR.TUMS.VCR.REC.1397.804).

### 2.2 Evaluation of anthropometric components

Participants’ anthropometric components including obesity degree percentage (ODP), fat‐free mass (FFM), visceral fat area (VFA), body fat percentage (BFP), and body fat mass (BFM) were determined by body composition analyzer (InBody770 scanner; InBody, Seoul, Korea). In this assessment, participants had to be free of any metal objects and without shoes or socks according to the instructions. Assessments were not performed after extreme exercise, individuals rested for at least twenty minutes before the measurements, and the evaluations were conducted 2 hr after the last meal (Mirzababaei et al., 2019). A digital scale (Seca, Hamburg, Germany) was used to measure weight, and height was evaluated by a seca stadiometer in a barefoot and light clothing condition. Hip circumference (HC) and waist circumference (WC) were assessed in the largest girth and the smallest girth, respectively. BMI was considered as a ratio of weight (kg) to height (m^2^).

### 2.3 Biochemical assessment

After 10–12 hr of fasting at night, a blood sample was taken and serum was isolated at −10℃ after centrifuging. Sample analysis was performed based on the manufacturer's protocol. Measurements were taken at the Biochemistry Laboratory of School of Health, Tehran University of Medical Sciences. Serum concentration of glucose by using a glucose oxidase method and insulin level by using an enzyme‐linked immunosorbent assay (ELISA) kit (Human insulin ELISA kit, DRG Pharmaceuticals, GmbH, Germany) was assessed. Total cholesterol (TC), triglyceride (TG), low‐density lipoprotein cholesterol (LDL‐C), and high‐density lipoprotein cholesterol (HDL‐C) were measured by Pars Azemun, Iran kits, autoanalyzer approach. hs‐CRP was assessed with the use of the immunoturbidimetric assay, IL‐1β was evaluated by IL‐1β Quantikine ELISA kit, R&D System, USA, and TGF‐β by HUMAN TGF‐BETA, and TAC was measured by ZellBio (cat. No: ZB‐TAC‐A96), GmBh (cat. No:ZB_TAC‐A48) kit.

### 2.4 Sleep quality assessment

For assessing the sleep quality over the women, PSQI was confirmed. This questionnaire includes 18 questions related to seven components including sleep quality, sleep disorders, sleep duration, delay in falling asleep, SE, use of sleeping pills, disturbances in daily activities due to poor sleep quality. Moreover, there are five questions in this questionnaire that are answered by someone who lives with the participant, which are not considered in scoring. The total score range is from 0 to 21 that resulting from seven components mentioned above, and each item has between 0 and 3 points. A score higher than five indicates poor sleep quality (Buysse et al., 1989). In Iran, the accuracy and validity of the PSQI questionnaire have been evaluated (Shahrifar, 2009).

### 2.5 LCD evaluation Calculation of the LCD score

Evaluation of LCD was performed by calculating the score of this diet. A semiquantitative FFQ containing a list of 147 Iranian foods agents plus their standard serving sizes, which assesses the usual dietary intake in the last year, was used for calculating of LCD score. The validity and reliability of this questionnaire have been approved (Esmaillzadeh et al., 2005). Participants were divided into 11 strata based on refined grains, carbohydrates, monounsaturated fatty acids (MUFA), n3/n6 polyunsaturated fatty acids (PUFA) intake, vegetable proteins that are expressed as a percentage of energy intake as well as fibers (gr/1000 Kcal), and glycemic load (GL). Dietary GL was calculated as (total glycemic index * total available carbohydrate)/100 (Wolever et al., 2006). Scoring is as follows: participants in the highest strata of refined grains, carbohydrates and GL were given a score of 0 and those in the lowest strata were given a score of 10 and for fiber, vegetable protein, MUFA, n3/n6 PUFA intake this approach was reversed. Total scores of these seven components were summed to calculate LCD score, which ranged from 0 (the lowest adherence of LCD which means the lowest fat and protein intake and the highest carbohydrate intake) to 70 (the highest adherence of LCD which means the highest protein and fat intake and the lowest carbohydrate intake) (Allebrandt & Roenneberg, 2008).

### 2.6 Other variables

#### 2.6.1 Demographic components

Demographic questionnaire was used to assess variables such as marital situation, employment situation, and education. According to the results of this questionnaire, individuals were divided into two groups in terms of marriage status: single and married, in terms of employment status into two groups of unemployed and employed, and in terms of educational status into three groups: illiterate, diploma, and university educated. It should be noted that we also considered the job as a confounder (as a quantitative variable).

#### 2.6.2 Stress assessment

Stress was considered as a cofounder variable. Depression anxiety stress scale (DASS) questionnaire with twenty‐one‐item self‐reported was confirmed for depression, anxiety, and stress evaluation in the last week. Calculating of the total scores for each subscale, a responder shows a four‐point scale with the degree according to which each item was applied during the last week, and also, *z*‐scores were used to define differences (Brown et al., 1997).

#### 2.6.3 Evaluation of physical activity

It was a cofounder variable in our study. International physical activity questionnaires, the complex questionnaires, that are specially used for research purposes were used for physical activity assessments. Nine items were used for weekly physical activity assessments according to metabolic equivalent (MET) scores for each item of activity (Craig et al., 2003; Moghaddam et al., 2012).

### 2.7 Statistical analyses

The normal distribution of data was checked by the Kolmogorov–Smirnov test. We used mean and standard deviation to describe quantitative variables and reported number and percentage for describing of qualitative data. Quartiles were made for assessing LCD score; then, we divided them into two groups: first which contained first and second quartiles and second which contained third and fourth quartiles. First group indicates low adherence of LCD and second group indicates high adherence of LCD. To assess the quality of sleep, individuals were divided into two groups according to the scores of the PSQI with cut points: 0–5 as good quality and 6 to 21 as poor quality. The relationship between sleep quality and LCD with quantitative variables was assessed by independent‐sample *t*‐test and with qualitative variables by Chi‐square test. In order to determine the difference between the means of the studied variables in the groups related to LCD and sleep quality, analysis of covariance (ANCOA) was used that adjusted for job, weight, and physical activity. Binary logistic regression was used to estimate confounding variables including age, job, stress, and weight to investigate the relationship between LCD and sleep quality in this modeling; the groups of “good sleep quality” and “low adherence of LCD” were considered as reference. In this study, the mediating role of inflammatory factors (hs‐CRP, TAC, IL‐1β, TGF‐β) in the relationship between LCD and sleep quality was also investigated that for this purpose, we entered each of these factors into the final model separately as a confounding variable, and each of them, which destroyed the significance of the model, was considered a mediating factor. Statistical analyses were performed using IBM SPSS version 25.0 (SPSS, Chicago, IL, USA). *p* value ≤ 0.05 was considered as acceptable level of significance.

## 3 RESULT

### 3.1 Characteristics of women studied

BMI ranged from 25.2 to 48.3 kg/m^2^ in the study population. In this investigation, the mean (*SD*) BMI, weight, height, age, LCD score, and sleep quality were, 30.83 (3.71) kg/m^2^, 77.81 (11.51) kg, 160.78 (6.02) cm, 35.69 (7.88) years, 33.98 (9.91), and 5.76 (3.55), respectively, and the number (percentage) marriage, job situation, and education were 230 (78%) single and 65 (22%) married, 95 (67%) unemployed and 96 (33%) employed, 43 (14.6%) illiterate and 87 (29.5%) diploma and 165 (55.9%) university educated; respectively. Table 1 compares the mean of quantitative variables between the two groups of LCD. Before ANCOVA test, in order to moderate the effect of confounding variables including job, weight, and physical activity, it was concluded that height (*p* =.001), weight (*p* =.02), FFM (*p* =.004), WC (*p* =.02), WHR (*p* =.005), job situation (*p* =.002), and LCD have a significant relationship, and after adjustment, a significant association between LCD and age (*p* =.04), height (*p* =.02), BMI (*p* =.02), ODP (*p* =.05), fat mass index (FMI) (*p* =.03), and the significance for job situation became stronger (*p* =.001). Also, there was a marginal significance with body weight (*p* =.06), TC (*p* =.08), LDL (*p* =.07), hs‐CRP (*p* =.07) and IL‐1β (*p* =.07). Age and FMI were higher in the high adherence group, and body weight, BMI, ODP, and both unemployed and employed groups were higher in the low adherence group. Only GL (*p* =.001) of LCD components had a significant relationship with LCD groups, the mean of which was higher in the lower adherence group (Table 1).

**TABLE 1:** Characteristics of individuals in LCD groups

Variable	Low adherence of LCD group (*n* = 243)	High adherence of LCD (*n* = 57)	*p*‐value^a^	*p*‐value ^**^
Age (years)	36.21 ± 8.47 ^1^	37.92 ± 8.59	0.18	0.**04**
weight (kg)	81.45 ± 12.49	77.38 ± 10.39	0.**02**	0.06
Height (cm)	161.83 ± 5.59	158.84 ± 6.78	0.**001**	0.**02**
Stress status	7.89 ± 5.01	8.28 ± 5.18	0.62	0.61
Physical activity (MET‐minutes/week)	1,153.45 ± 1778.2	1,413.54 ± 3,173.56	0.44	0.42
Blood parameters				
FBS (mmol/dl)	87.5 ± 8.88	87.85 ± 12.64	0.82	0.3
TC (mg/dl)	183.6 ± 36.11	190.65 ± 37.04	0.23	0.08
TG (mg/dl)	122.77 ± 73.28	119.53 ± 57.4	0.77	0.75
HDL (mg/dl)	46.81 ± 11.02	46.46 ± 10.32	0.84	0.82
LDL (mg/dl)	93.99 ± 24.23	98.17 ± 25.36	0.29	0.07
hs‐CRP (mg/L)	4.1 ± 4.49	4.84 ± 4.95	0.32	0.07
TGF‐β (mg/L)	77.57 ± 48.42	81.41 ± 49.32	0.68	0.4
IL−1β (mg/L)	2.64 ± 0.95	3.01 ± 0.82	0.52	0.07
TAC(µmol/L)	440.37 ± 88.74	428.49 ± 120.37	0.18	0.86
Body composition				
BMI (kg/m^2^)	31.11 ± 4.29	30.77 ± 4.47	0.6	0.**02**
BFM (kg)	34.38 ± 8.78	32.28 ± 7.99	0.11	0.78
BFP (%)	41.5 ± 5.47	41.59 ± 5.89	0.91	0.39
FFM (kg)	47.21 ± 5.58	44.79 ± 5.14	0.**004**	0.24
FFMI	17.98 ± 1.53	17.76 ± 1.37	0.36	0.19
FMI	13.14 ± 3.29	13.19 ± 3.84	0.92	0.**03**
WC (cm)	99.57 ± 10.18	96.17 ± 8.94	0.**02**	0.8
WHR	0.93 ± 0.05	0.91 ± 0.04	0.**005**	0.19
VFA (cm)	164.79 ± 38.61	155.6 ± 37.63	0.12	0.96
ODP	144.34 ± 20.44	143.11 ± 21.04	0.69	0.**05**

Other variables							
		*N*	%	*N*	%	*p* value***	*p* value**
Marriage status	Single	190	81.8	42	18.2	0.46	0.4
	Married	53	76.8	15	23.2		
Job situation	Unemployed	172	85.6	28	14.4	0.**002**	0.**001**
	Employed	71	70.6	29	29.4		
Education	Illiterate	38	82	8	18	0.1	0.11
	Diploma	79	86.7	12	13.3		
	University educated	126	76.5	37	23.5		

Note

*p*‐values are resulted from independent‐sample *t*‐test. ^**^ *p*‐value reported after adjusting weight, physical activity and job with ANCOVA model. ***p‐values are resulted from Chi‐square test.

Abbreviations: 1, Mean ± *SD*; BFM, body fat mass; BFP, body fat percentage; BMI, body mass index; FBS, fasting blood sugar; FFM, fat‐free mass; FFMI, fat‐free mass index; FMI, fat mass index; HDL, high‐density lipoprotein; hs‐CRP, high‐sensitivity C‐reactive protein; Il‐1β, interleukin‐1β; LDL, low‐density lipoprotein; *N*, 304; ODP, obesity degree percentage; TAC, total antioxidant capacity; TC, total cholesterol; TG, triglyceride; TGF‐β, transforming growth factor β; VFA, visceral fat area; WC, waist circumference; WHR, waist hip ratio.

In Table 2, we compared the mean of quantitative variables between the two groups of sleep quality. Before ANCOVA test, in order to moderate the effect of confounding variables including job, weight, and physical activity, it was concluded that stress status (*p* <.001), TGF‐β (*p* =.02), and marriage status (*p* =.02) were significantly associated with sleep quality, and after adjustment, the relationship for stress status (*p* <.001), TGF‐β (*p* =.02) and marriage status (*p* =.02) remained significant and other variables including WHR (*p* =.03) and WC (*p* =.05) became significantly correlated with sleep quality. Single and married had a higher percentage in poor sleep quality group, and also other variables had a higher mean in poor sleep quality group. None of the LCD components were related to sleep quality (Table 2).P‐value≤ 0.05 is significant

**TABLE 2:** Description of characteristics among groups of sleep quality

Variable	Good quality (*n* = 86)	Poor quality (*n* = 218)	*p* value^b^	*p*‐value**
Age (years)	36.56 ± 8.45^1^	36.05 ± 8.86	0.66	0.53
weight (kg)	79.98 ± 12.6	80.41 ± 11.69	0.79	0.92
Height (cm)	161.13 ± 6.12	161.12 ± 5.85	0.98	0.84
Stress status	6.45 ± 4.43	9.59 ± 5.14	**<0.001**	**<0.001**
Physical activity (MET‐minutes/week)	961.64 ± 1,014.94	1,241.23 ± 1983.29	0.24	0.44
Blood parameters				
FBS (mmol/dl)	87.63 ± 9.57	86.79 ± 9.68	0.55	0.98
TC (mg/dl)	184.19 ± 30.74	176.97 ± 33.46	0.12	0.34
TG (mg/dl)	126.68 ± 64.18	113.9 ± 62.59	0.17	0.34
HDL (mg/dl)	47.7 ± 9.88	46.2 ± 9.27	0.29	0.49
LDL (mg/dl)	100.34 ± 22.41	95.33 ± 22.67	0.13	0.42
Hs‐CRP (mg/L)	4.87 ± 5.05	4.05 ± 4.53	0.24	0.49
TGF‐β (mg/L)	71.95 ± 35.7	87.84 ± 41.03	0.**02**	0.**02**
IL−1β (mg/L)	2.53 ± 0.67	2.79 ± 1.25	0.35	0.56
TAC (µmol/L)	442.78 ± 97	3.66 ± 6.6	0.53	0.54
Body composition				
BMI (kg/m^2^)	30.7 ± 4.22	31.05 ± 4.31	0.54	0.88
BFM (kg)	33.28 ± 8.48	34.13 ± 8.71	0.46	0.42
BFP	41.25 ± 5.31	41.86 ± 5.4	0.4	0.5
FFM	46.58 ± 6.14	46.38 ± 4.96	0.78	0.47
FFMI	17.88 ± 1.56	17.84 ± 1.38	0.84	0.25
FMI	12.85 ± 3.26	13.24 ± 3.42	0.39	0.57
WC (cm)	97.74 ± 10.33	98.93 ± 9.62	0.37	0.**05**
WHR	0.92 ± 0.05	0.93 ± 0.05	0.17	0.**03**
VFA (cm)	158.7 ± 38.77	164.45 ± 38.11	0.27	0.15
ODP	142.95 ± 19.73	144.39 ± 20.09	0.59	0.96

Other variables							
		*n*	%	*n*	%	*p*‐value***	*p*‐value**
Marriage status	Single	50	40.9	132	59.1	0.**02**	0.**02**
	Married	36	41.8	86	58.2		
Job situation	Unemployed	52	40.3	147	59.7	0.31	0.28
	Employed	34	48.1	71	51.9		
Education	Illiterate	20	43.5	34	56.5	0.9	0.65
	Diploma	29	44.7	74	55.3		
	University educated	37	41.8	110	58.2		

Note

*p‐values are resulted from independent‐sample *t* test. **p‐value reported after adjusting weight, physical activity and job with ANCOVA model. ***p‐values are resulted from Chi‐square test.

Abbreviations: 1, Mean ± *SD*; BFM, body fat mass; BFP, body fat percentage; BMI, body mass index; FBS, fasting blood sugar; FFM, fat‐free mass; FFMI, fat‐free mass index; FMI, fat mass index; HDL, high‐density lipoprotein; hs‐CRP, high‐sensitivity C‐reactive protein; Il‐1β, interleukin‐1β; LDL, low‐density lipoprotein; *N*, 304; ODP, obesity degree percentage; TAC, total antioxidant capacity; TC, total cholesterol; TG, triglyceride; TGF‐β, transforming growth factor β; VFA, visceral fat area; WC, waist circumference; WHR, waist hip ratio. P‐value≤ 0.05 is significant

### 3.2 The association of the LCD and sleep quality

In crude model, there was no significant relationship between LCD and sleep quality (OR: 0.61, 95% CI: 0.3 to 1.21, *p*: 0.16). In the final model, after adjusting the confounding variables including job, age, stress, and weight, the relationship between high LCD adherence and sleep quality became significant (OR =0.43, 95% CI =0.19 to 0.94, *p* =.03). We found that by increasing the following LCD compared to low adherence of it, the odds of poor sleep quality compared to good sleep quality decreased (Table 3).

**TABLE 3:** Relation between adherence of LCD and sleep quality

	Sleep quality**				
			OR(95% CI)	β ± SE	*p*‐value
Crude model	Low LCD adherence		1*	1	0.16
	High LCD adherence		0.61(0.3–1.21)	−0.49 ± 0.35	
Model 1^a^	Low LCD adherence		1	1	0.03
	High LCD adherence		0.43(0.19–0.94)	−0.83 ± 0.4	
Model 2^b^	Hs‐CRP	High LCD adherence	0.61(0.3–1.21)	−0.49 ± 0.35	0.16
	TGF‐β	High LCD adherence	0.43(0.19–0.94)	−0.83 ± 0.4	0.03
	IL−1β	High LCD adherence	0.14(0.02–0.99)	−1.94 ± 1.00	0.05
	TAC	High LCD adherence	0.6(0.25–1.4)	−0.5 ± 0.43	0.24

Notes

*Low LCD adherence is considered as reference. **In sleep quality categorization “good quality” consider as reference.

Abbreviations: binary logistic regression; CI, confidence interval; crude model and adjusted model to age, job, weight, stress as covariates; LCD, low carbohydrate diet; *N*, 304; OR, odds ratio; SE, standard error.

^a^ Modeling without considering the mediating effect of inflammatory factors.

^b^ Modeling by considering the mediating effect of inflammatory factors. P‐value≤ 0.05 is significant

### 3.3 The association of mediatory effect of inflammatory markers

In this study, the mediating role of inflammatory factors (hs‐CRP, TAC, IL‐β, TGF‐β) in a significant relationship between LCD and sleep quality was investigated. The results of previous investigations have found that eating less carbohydrate can reduce some inflammatory markers such as IL‐6, hs‐CRP, and IL‐8, and it can increase the concentration of adiponectin as an anti‐inflammatory marker(Due et al., 2005; Waldman et al., 2020), and also, the results of our analyses showed that there was no significant relationship between other dietary ingredients and inflammatory markers. Therefore, we decided to choose the LCD for evaluation. Finally, we found that two inflammatory factors, hs‐CRP (OR =0.61, 95% CI =0.3 to 1.21, *p* =.16) and TAC (OR =0.6, 95% CI =0.25 to 1.4, *p* =.24), destroyed the significant relationship between LCD and sleep quality, so they were considered as mediating factors in this relationship (Table 3).

## 4 DISCUSSION

As far as we know, our investigation is the first investigation that has examined the relationship between LCD and sleep quality mediated by inflammatory factors (hs‐CRP, TAC, IL‐1β, TGF‐β) in the problem of overweight and obesity among women.

It was showed that LCD was significantly associated with sleep quality. Our findings suggested that increasing LCD scores or intake of LC can reduce the odds of poor sleep quality by 43% compared to good sleep quality group. In fact, a negative association between LCD scores and the PSQI scores was found. Moreover, inflammatory factors, TAC, and hs‐CRP may have mediatory roles in this correlation. A significant association between sleep quality and variables including TGF‐β, WC, WHR, and marriage status was found. They were positively correlated with PSQI scores, and their means were higher in poor quality group. So, it may be found that individuals with sleep quality disorders had a higher odd for becoming obese. However, a significant correlation between LCD and age and FMI was also found that they were higher in the low adherence group and height, BMI and ODP and job situation that were higher in the low adherence group. So, participants that had higher scores of LCDs may had a lower risk of being obese.

Findings show that the LCD produced clinically significant reductions in body weight (6% from baseline), LDL cholesterol, blood pressure, and fasting insulin levels (Kalam et al., 2021). One study concluded that patients who consumed lower carbohydrate have better sleep status (Daneshzad et al., 2020). Poor sleepers with overweight or obesity may become good sleepers when following LCD energy‐restricted diets (Hudson et al., 2020). Different studies have demonstrated a relationship between obesity and sleep duration and its complications like diabetes and cardiovascular diseases (Cappuccio et al., 2007, 2010; Chao et al., 2011). In one study, BMI, WC, and BFP were related to sleep quality score( Jennings et al., 2007). Rohit Sane et al. have found that LCD, along with other weight loss programs, can reduce BMI, weight, and WC (Sane et al., 2019). Kwan et al. investigated the effect of LCD contains 50 g/d of carbohydrate during a week, on sleep quality among the women. REM‐onset latency increased after the LCD relative to habitual diet (Kwan et al., 1986). A study showed that having a HC meal can reduce non‐REM sleep and also increases the number of REM periods compared to a free or low carbohydrate treatment over the entire sleep period, and carbohydrate oxidation was highest after HC meals and during REM sleep (Porter & Horne, 1981). A study concluded that eating higher carbohydrate foods such as confectionary, noodles, rice, also sugar‐sweetened beverages was associated with poor sleep quality, as evidenced by a high global PSQI score, whereas a high intake of fish was associated with good sleep quality. A significant correlation between worse sleep quality and increasing carbohydrate intake was found (Katagiri et al., 2014). Many studies have shown that short sleep duration can lead to insulin resistance, which is a key factor in the pathophysiology of metabolic syndrome (Marc‐Andre et al., 2008). Sleep limitation may increase inflammatory markers such as hs‐CRP (Meier‐Ewert et al., 2004). Insufficient sleep can increase food intake through organizing some neuroendocrine, for example, changes in leptin and ghrelin concentrations and other metabolic and behavioral compatibility so weight gain and inflammatory status such as obesity may occur (Mullington et al., 2009; Penev, 2012). LCD can reduce fasting blood glucose concentration and glycogen synthesis and storage. Therefore, the availability of glucose in organs such as brain, liver, and muscle will be decreased. After limited access to glucose, different endocrine changes occur over the body that lead to the production of ketone bodies. So, insulin release will be decreased, and also glucose and fat storage will be reduced, consequently. By reducing the amount of fat cells storage, the pro‐inflammatory marker release and adipokines synthesize may be limited as a result (Hall, 2017; Van Wyk et al., 2016). Studies have shown that dietary carbohydrate restriction reduces reactive oxygen species (ROS) generation by polymorphonuclear leukocytes and mononuclear cells and inflammatory markers such as IL‐6, hs‐CRP, and IL‐8 and increases the concentration of adiponectin as an anti‐inflammatory marker (Due et al., 2005; Waldman et al., 2020). If the amount of protein intake in the LCD is higher than routine (sometimes the amount of protein intake in this dietary pattern is higher than routine and sometimes not), higher amount of tryptophan consumption, which contains milk and some other foods, can improve the quality of sleep (Bhatti et al., 1998). The mechanism is that tryptophan, as a precursor to the synthesis of serotonin in the brain, competes with other amino acids to cross the blood–brain barrier, and serotonin, as a precursor to the synthesis of melatonin, the sleep‐inducing hormone, is involved in sleep regulation. Some studies have shown that high concentrations of melatonin and serotonin resulted to improvements in sleep quality (Garrido et al., 2013). For the first time in Iran, we studied the relationship between LCD and sleep quality mediated by inflammatory factors, which may be considered as a new approach in the treatment of obesity. We examined the relationship of anthropometric components and biochemical parameters and sleep quality, as well as LCD over the study. In addition, we selected inflammatory markers as mediatory factors that have not been considered in previous studies. We targeted women because the study of this group is not enough, so we decided to take steps to improve women's health as one of the most sensitive groups in society. We also tried to include socioeconomic factors among the confounding variables because sleep quality is strongly associated with these factors. Briefly, this study is a cross‐sectional study so causal inferences could not be extracted only by correlation. There may be more socioeconomic factors affecting the sleep quality. LCD evaluation was performed by FFQ that over‐reporting and under‐reporting errors are common in this method.

## 5 CONCLUSION

In the present evidence, there is a significant association between LCD adherence and PSQI scores and according to the results of the study adherence of this dietary pattern reduced PSQI scores that indicate good sleep quality in overweight and obese women. Based on our studies, two inflammatory factors (TAC and hs‐CRP) were considered as possible mediating factors in this association.

## 6 PARTICIPANT CONSENT

Subjects signed a written informed consent.

## CONFLICTS OF INTEREST

The authors declare that there is no conflict of interest in this study.

## AUTHOR CONTRIBUTIONS

**Atefeh Tavakoli:** Conceptualization (equal); Investigation (equal); Methodology (equal); Writing‐original draft (equal). **Atieh Mirzababaei:** Data curation (equal); Writing‐review & editing (equal). **Khadijeh Mirzaei:** Supervision (equal).

## ETHICAL APPROVAL

The study protocol was approved by the ethics committee of Tehran University of medical sciences (IR.TUMS.VCR.REC.1397.804) and is acknowledged by authors. All participants signed a written informed consent.

## Data Availability

The data that support the findings of this study are available from Dr. Khadijeh Mirzaei but restrictions apply to the availability of these data, which were used under license for the current study, and so are not publicly available. Data are, however, available from the authors upon reasonable request and with permission of Dr. Khadijeh Mirzaei.
